# Young Adults View Smartphone Tracking Technologies for COVID-19 as Acceptable: The Case of Taiwan

**DOI:** 10.3390/ijerph18031332

**Published:** 2021-02-02

**Authors:** Paul M. Garrett, YuWen Wang, Joshua P. White, Shulan Hsieh, Carol Strong, Yi-Chan Lee, Stephan Lewandowsky, Simon Dennis, Cheng-Ta Yang

**Affiliations:** 1 School of Psychology, University of Melbourne, Melbourne 3010, Australia; paul.garrett@unimelb.edu.au (P.M.G.); josh.white@unimelb.edu.au (J.P.W.); Simon.Dennis@gmail.com (S.D.); 2 Department of Psychology, National Cheng Kung University, Tainan 701, Taiwan; r06227122@g.ntu.edu.tw (Y.W.); psyhsl@mail.ncku.edu.tw (S.H.); 3 Institute of Allied Health Sciences, National Cheng Kung University, Tainan 701, Taiwan; 4 Department of Public Health, National Cheng Kung University, Tainan 701, Taiwan; carolcj@mail.ncku.edu.tw; 5 Department of Otolaryngology - Head and Neck Surgery, Chang Gung Memorial Hospital, Keelung 114, Taiwan; b9002063@cgmh.org.tw; 6 School of Psychology, The University of Bristol, Bristol BS8 1TU, UK; stephan.lewandowsky@bristol.ac.uk; 7 Unforgettable Research Services, Melbourne 3010, Australia

**Keywords:** COVID-19, tracking technologies, SARS-CoV-2, contact tracing, Taiwan, public health, health policy, privacy, privacy calculus

## Abstract

Taiwan has been successful in controlling the spread of SARS-CoV-2 during the COVID-19 pandemic; however, without a vaccine the threat of a second outbreak remains. Young adults who show few to no symptoms when infected have been identified in many countries as driving the virus’ spread through unidentifiable community transmission. Mobile tracking technologies register nearby contacts of a user and notifies them if one later tests positive to the virus, potentially solving this issue; however, the effectiveness of these technologies depends on their acceptance by the public. The current study assessed attitudes towards three tracking technologies (telecommunication network tracking, a government app, and Apple and Google’s Bluetooth exposure notification system) among four samples of young Taiwanese adults (aged 25 years or younger). Using Bayesian methods, we find high acceptance for all three tracking technologies (>75%), with acceptance for each technology surpassing 90% if additional privacy measures were included. We consider the policy implications of these results for Taiwan and similar cultures.

## 1. Introduction

**Figure 1:**
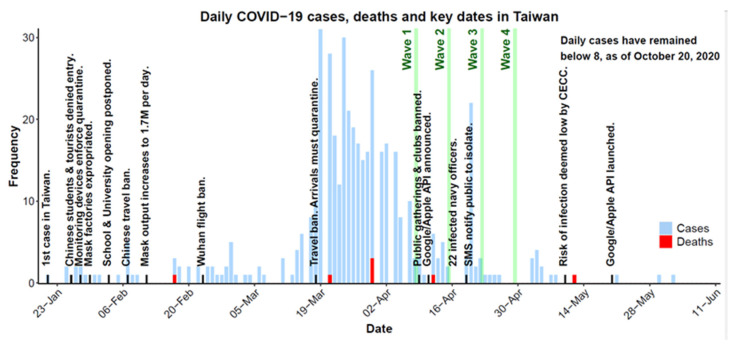
COVID-19 daily cases (blue), deaths (red), and key policy decisions (text) in Taiwan during the COVID-19 pandemic within the period 23 January–11 June 2020. Collection dates of the current study are highlighted in green. In the time between 11 June and 20 October 2020, Taiwan recorded a further 95 cases and a maximum of seven cases per day. CECC—Central Epidemic Command Center. API—Application Programing Interface.

Despite its proximity and close cultural and economic ties to China, Taiwan has managed to arrest the spread of the severe acute respiratory coronavirus 2 (SARS-CoV-2) disease responsible for the COVID-19 pandemic. Proactive policy decisions informed by the Central Epidemic Command Centre (see Figure 1) [1], combined with public health behaviors like hand washing, social distancing [2], and mask wearing [3], have limited the virus’ impact to 540 total cases and 7 deaths in a population of 23.7 million (as of 20 October 2020) [4]. However, the highly transmittable and often asymptomatic nature of the virus [5] means Taiwan may yet face another COVID-19 outbreak. Policy makers must be informed of which non-pharmaceutical measures may be implemented without halting the nation’s social and economic activities, and if these measures are likely to be effective among those individuals most likely to spread the virus: young adults [6].

Young adults who frequently engage in social activities and present with little to none of the SARS-CoV-2 symptoms [7] have been identified as driving the spread of COVID-19 in many countries [6]. Unknowingly, these individuals may act as “superspreaders” [8], and appear to have played a role in the second and third waves of infections already impacting some countries (e.g., Australia, the United Kingdom, and the United States of America) [9]. Manual contact tracing is an important and time-intensive process [10] that relies on individuals remembering their location and contact history.

Subsequently, these efforts may overlook community transmissions that occur between young adults at public events or on public services, thereby allowing the virus to spread unfettered. Mobile tracking technologies provide one method for rapidly identifying community transmissions and reducing the likelihood of a second outbreak among young Taiwanese adults, while allowing policy makers to maintain social and economic activity. The following study identifies the risks, benefits, and acceptance of tracking technologies for COVID-19 among young Taiwanese adults. 

### 1.1. Tracking Technologies

Mobile tracking technologies use GPS, Bluetooth, or telecommunication network data to identify a list of contacts with whom you have been closely located [11]. These contacts are either stored on a centralized server accessible by manual contact tracers working in the health department (centralized system), or locally on the user’s phone (decentralized system). If a contact later tests positive to COVID-19, their contacts will receive either phone-to-phone notifications (decentralized) or be notified by contact tracers working in the health department (centralized) [12].

Policy makers must understand the perceived benefits and risks associated with these technologies if they wish to gain the social license (i.e., broad public acceptance) [13] for their introduction. When disclosing potentially private information, people tend to weigh the benefits (public health) against the risks (data surveillance) in an internal “privacy calculus” [14,15,16]. Here, we describe privacy risks in terms of the uncertainty caused by the possible misuse of sensitive or private information [17]. With some technologies requiring vast public uptake to be effective (minimum tracking app uptake may be as high as 60% of the population) [18], public policy must aim to maximize acceptance by ensuring these perceived benefits outweigh these perceived risks.

Evaluating the perceived risk–benefit profiles of a new health policy can be aided by the framework of the Health Belief Model [19,20]. This model identifies six critical factors underlying successful public health policies: perceived illness susceptibility and severity, policy benefits and barriers, cues to action, and self-efficacy [19]. Putting aside cues to action (e.g., government advertising) for now, the remaining factors may be readily identified for COVID-19 tracking technologies.

Using the Health Belief Model, we identified perceived COVID-19 susceptibility and severity (COVID-19 risk), perceived tracking benefits (one’s own and others’ health), perceived tracking risks (data security and privacy), and control (self-efficacy regarding when tracking occurs and who has data access) as critical factors to gaining public acceptance for COVID-19 tracking technologies. The risks posed by COVID-19 will vary with the state of the pandemic (increasing or decreasing cases), and so too may the relative benefits and risks associated with each technology (more beneficial when cases are high). However, some technological features—data access and data control—may have a stable public perception regardless of COVID-19 cases.

Table 1 displays how mobile tracking technologies vary in their risk–benefit profiles. GPS locations are precise, but cannot be reliably detected indoors [21] where the virus is likely to spread. Telecommunication network tracking is used as part of the Taiwan’s “electronic fence” to monitor individual’s movements under home-quarantine [22,23] and could be adapted to collocate individuals, however, location precision varies with the availability of network towers [24]. Bluetooth tracking works in a limited radius [25] and can preserve privacy through anonymous phone IDs, but only works if both users have downloaded compatible COVID-19 tracing apps [12,26]. Similarly, data storage systems offer benefits and risks: a decentralized system ensures data privacy and security, but at a cost to assisting manual contact tracing efforts. Understanding how young Taiwanese adults trade-off these risks and benefits when deciding if a policy is “acceptable” is critical to ensuring policy makers don’t waste time and resources pursuing ineffective solutions.

### 1.2. Broader Implications

Establishing acceptance for tracking technologies will of course benefit Taiwan, but may also benefit other countries that are culturally similar. Taiwan has shown different attitudes to Western countries regarding privacy attitudes [27], their communal sense of self [28], and approaches to governmental public health surveillance (e.g., compare Taiwan’s rapid COVID-19 response and the sharing of public health records, to the response by America, the United Kingdom, or Australia) [1]. These differences may make the introduction of tracking technologies more effective in Taiwan and countries with similar cultures, as public health may be viewed as more important than individualistic notions of privacy and security [29]. As policy makers around the world seek solutions to the spread of COVID-19, it is important that research reflects these diverse attitudes, as a health policy that works in one country may not work in another.

To these ends, the current research fits within a broader series of investigations determining acceptance for non-pharmaceutical solutions to stopping COVID-19. Currently, this research sits at the vanguard of a series of ongoing investigations in the United Kingdom [30], Australia [31], the United States of America, Germany, Hungary, Switzerland, Italy, Spain, and Japan, preliminary analyses of which can be found at https://stephanlewandowsky.github.io/UKsocialLicence.

### 1.3. Current Study

In April 2020, we asked a sample of young Taiwanese adults about their attitudes to introducing three hypothetical mobile tracking technologies: telecommunication network tracking, a centralized government app, and the Apple and Google exposure notification (EN) Bluetooth system (more on this in the methods). We also assessed their perceptions of the COVID-19 pandemic in Taiwan, and how acceptance for tracking technologies changed with the inclusion of additional measures that increased the user’s privacy, security and data control (e.g., a “Sunset” clause, local storage or opt-out option). This study aims to inform policy decision makers about the effectiveness of introducing COVID-19 tracking technologies in Taiwan and other Asian countries with similar attitudes towards health surveillance, and the factors that most improve acceptance for these technologies among young adults.

## 2. Materials and Methods

### 2.1. Overview

Four survey waves were collected one week apart during the COVID-19 pandemic in Taiwan between 8 April and 29 April 2020 (see Figure 1). Surveys assessed the attitudes of young Taiwanese adults to one of three mobile tracking technologies, in addition to a range of constructs such as world views, trust in government, and the impact and perception of COVID-19. Chinese and English versions of the surveys, data, and analysis code can all be accessed at our Open Science Framework (OSF) repository, https://osf.io/tcqae.

Waves one and two examined two hypothetical tracking scenarios. The first was a voluntary centralized government app that notified users if they were in contact with an infected individual and shared contact registries with the health department to allow manual contact tracing (“Gov App” scenario). The second was telecommunication network tracking through which the government could issue quarantine orders and fines (“Telecommunication” scenario). These items were developed when a government app had not been considered in Taiwan. Therefore, the tracking technology used by the government app was left ambiguous although the scenario was phrased to intimate the use of GPS or Bluetooth technologies. Participants were always made aware that the proposed technologies were hypothetical in nature.

Waves three and four included an additional “Bluetooth” scenario describing the newly introduced EN system proposed by Apple and Google. In this scenario, phones exchanged contact information through a decentralized system, directly notifying a user’s phone if a registered contact later identified as infected [26]. This scenario was added following Apple and Google’s announcement to create an EN system (11 April 2020), and data were collected before the app was launched (20 May 2020; see Figure 1). The EN system has a notable advantage in that any app developed with the EN system can share contact registry information. In this way, apps developed by different countries may still communicate with one another.

### 2.2. Participants

Participants were 1087 Taiwanese adults aged 18 to 25 who completed a 10 min (waves 1–2) or 15 min (waves 3–4) online survey, collected between 8 April and 29 April 2020. Between these dates, cumulative COVID-19 cases increased from 376 to 429, and deaths increased from five to six [4]. Participants were recruited through the online survey distribution platform, Surveycake, and were reimbursed NTD $50 or could optionally be reimbursed one course credit if they were a student of introductory psychology at the National Cheng Kung University. Ethics approval for this study was obtained from the Ethics Committee of the Department of Psychology at the National Cheng Kung University (ethics code 108-072). The study was conducted in accordance with the approved guidelines and regulations. Due to the rapidly evolving nature of the pandemic, as many participants were sampled as possible given immediate financial constraints and the need for rapid, informative data at the start of the pandemic.

### 2.3. Design and Procedure

**Figure 2:**
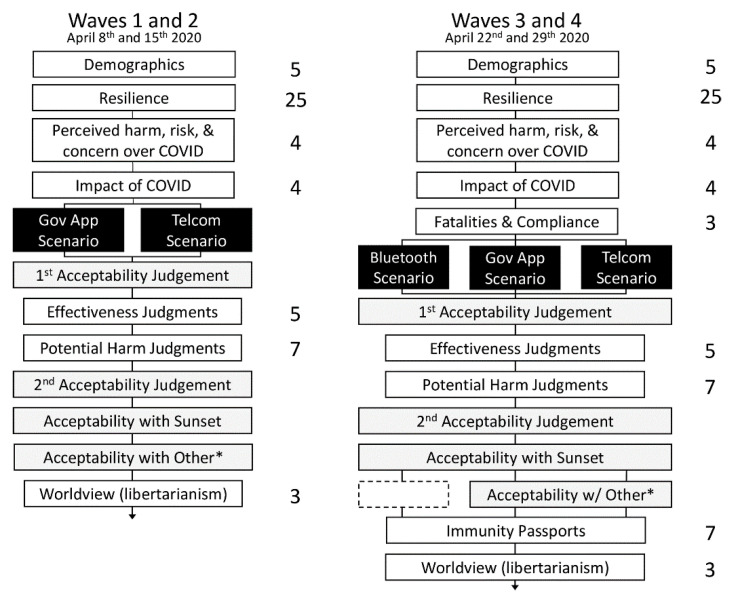
Survey design for waves 1–4. White boxes depict a block of questions with the number of items displayed on the right. Black boxes display the scenario to which participants were randomly assigned in a between-subjects design. Gray boxes illustrate judgments of tracking acceptability. “Acceptability with other*” included a local phone data-storage option for the government app scenario and the ability to opt-out of tracking in the telecommunication scenario.

Figure 2 illustrates the survey design in each wave of data collection. Plain language statements, consent, comprehension checks directly following each scenario, and free-text responses at the end of the surveys are not reported. Immunity passports (waves 3 and 4) will not be discussed in the current paper, and resilience and world view items will only be presented as an appendix, proving among the least important items examined in this study. 

After reading an information sheet and providing informed consent and demographic information, participants were assessed for their psychological resilience using the Connor–Davidson resilience scale (CD-RISC) [32]. This measure and associated results are presented in Appendix A. Following this, participants reported their perceived risk from COVID-19 (items summarized in Table 2). Responses were made on a 5-point scale, where higher items corresponded to an endorsement of the issue, (e.g., 1 = Not at all, 5 = Extremely). Participants were then probed about the impact of COVID-19 (Table 2), and in waves 3 and 4 were asked to estimate national fatalities and policy compliance. National fatality estimates were recorded to compare fatality estimates in other countries that were examining this item and will not be reported in the current study.

Participants were then presented with one of three tracking scenarios. Tracking scenarios are displayed in English and Chinese in Appendix B, and are summarized below. The government app scenario described a voluntary, centralized COVID-19 tracking app. Data would be stored in an encrypted format on a secure server accessible only to the Taiwanese government, and would only be used to contact those who might have been exposed to COVID-19.

The telecommunication network tracking scenario described mandatory mobile tracking with no possibility to opt-out. Data would be stored in an encrypted format on a secure server accessible only to the Taiwanese government who may use the data to locate people who were violating lockdown orders and enforce these orders with fines and arrests where necessary (i.e., an extension to the “electronic fence” system already in use).

The Bluetooth (i.e., Apple and Google’s EN system) scenario described a voluntary, decentralized contact tracing app that would use Bluetooth to help inform people if they have been exposed to others with COVID-19. The government would not know the identities of these individuals.

After reading one of the three scenarios, participants then completed a comprehension check, selecting an accurate scenario description from a range of choices. Participants who identified an incorrect description were excluded at analysis.

Tracking acceptability was then assessed after reading the scenario (1st acceptability) and then again after responding to items querying the benefits and risks posed by the technologies (2nd acceptability). This second measure was taken as the primary “acceptance” metric, as public acceptance in the “real world” is likely to change after considering the implications of these technologies.

To assess acceptability, participants were asked if they “would download and use” the government app, if “the use of [telecommunication network] tracking data in this scenario is acceptable”, and if they “would use” the Apple and Google Bluetooth technology. Items assessing the benefits and harm posed by each scenario are summarized in Table 3. Responses were made on a 6-point scale (1 = Not at all, 6 = Extremely).

Two follow-up items were asked if participants responded “no” to the second acceptability judgement. The first item asked if tracking would be acceptable under a sunset clause where data were deleted after 6 months. The second item asked if tracking would be acceptable if participants could opt-out of tracking (telecommunication network tracking scenario only), or if data were only stored locally on the phone rather than on a government server (government app scenario only). A second question was not asked in the Bluetooth scenario.

Acceptability items were then followed by items assessing attitudes to introducing immunity passports (waves 3 and 4) and political world views. World view items and results are displayed in Appendix A and immunity passport items will not be discussed in the current paper. 

### 2.4. Data Analysis

Treating Likert-style responses (ordinal variables) as interval data assumes that the perceived intervals between categories are evenly spaced (e.g., that the perceived interval between responses “none” and “slightly”, is equivalent to the interval between “very” and “extremely”). When violated, this assumption may undermine or invalidate parametric analyses that treat ordinal data as if it were continuous. To bypass this issue, we chose to model the latent categorical boundaries that underlie the ordinal responses using Bayesian methods. Latent ordinal boundaries were estimated from a single fixed boundary location (the lowest boundary) across participants and items, allowing for responses to be directly compared across items. This allows readers to interpret differences within the data using both qualitative boundaries (Likert response categories) and statistically meaningful Bayesian posterior estimates. Bayesian ordinal probit regressions were used to compare Likert-style responses using the MCMCoprobit and HPDinterval functions in R packages MCMCpack [33] and Coda [34], respectively. The Bayesian ordinal probit model assumes that latent normally distributed variables underlie the ordinal responses, and divides the latent space using one minus the number of response-option boundaries. Latent variables are then modelled with reference to the lowest boundary which is fixed at zero (following the method of [35]). Similar items and different scenario conditions were modelled together to allow direct comparisons between variables with the same response option boundaries between them (this will become clear in the results section; see [36] for model details).

To estimate a latent variable, the Bayesian model samples a posterior distribution of plausible means by weighing the likelihood of an observation against its prior probability of occurring in the sample. These normally distributed samples follow parametric assumptions and constrain the effect of outliers in the sampled data, allowing the densest and most credible region of data to be plotted and used to inform policy decisions. Practically, this means that instead of testing a threshold of significance (e.g., *p*-value), we may directly compare the 95% highest density credible intervals to determine differences in the data.

The MCMCoprobit function was run with one chain of 20,000 Markov Chain Monte Carlo (MCMC) iterations (including 1000 burn-ins) per variable, and a tuning parameter of 0.3 (the size of the Metropolis–Hastings step). Default uniform improper priors over real numbers between -inf to inf were used for the mean and the boundary parameters.

Highest density intervals were determined for bimodal variables (e.g., binary “yes” or “no” responses) using the bayes.prop.test function from the BayesianFirstAid package [37]. The bayes.prop.test function was run with one chain and 20,000 MCMC interactions (including 1000 burn-ins). Default priors were used: a beta distribution (α = 1, β = 1) corresponding to a uniform prior over the unit interval. Ninety-five percent highest posterior density intervals (HDIs) were estimated on the resulting posterior samples.

## 3. Results

Anonymized data and analysis code are available for all waves at https://osf.io/tcqae. As the reader will soon see, perceptions regarding COVID-19 were relatively stable across the waves of data collection and there were relatively few new cases of COVID-19 during this collection period (increasing from 376 to 429). As such, we chose to report our main findings collapsed across the four waves of data collection.

### 3.1. Data Preparation and Demographics

Participants under the age of 18 or older than 25, participants that failed to pass a comprehension check, or participants that did not complete the survey were removed from each sample (see Table 4). The final sample retained across the waves was N = 957. Demographic information is provided in Table 4. Across waves 2–4, 93% of participants owned a smartphone.

### 3.2. Impacts of COVID-19

Across all four waves, 96% of participants reported as being under lockdown for an average of 0 (SD = 2) days. Of the 957 participants sampled, 21 (2%) lost their job due to COVID-19, 2 participants tested positive with COVID-19 and 9 participants knew someone who had tested positive to COVID-19. In the final two waves, participants were asked to estimate public compliance with government COVID-19 policy decisions, for example, lockdown laws and social distancing. In wave 3 compliance was estimated to be 41% and in wave 4 compliance was estimated at 44%.

### 3.3. Perceived Risk from COVID-19

**Figure 3:**
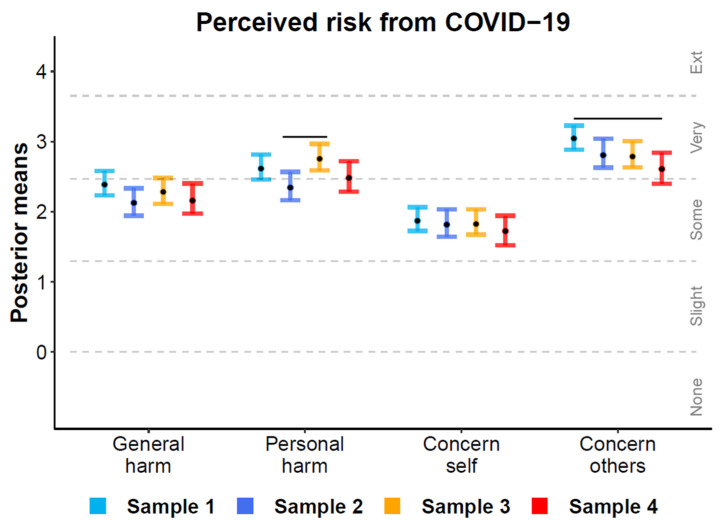
Ordinal regression mean posterior distributions for items assessing the perceived risk from COVID-19 for each wave. Black points display posterior means and colored error bars display the 95% highest posterior density interval. Dotted lines depict boundaries separating the latent space into ordinal responses (none to extremely). Non-overlapping intervals within items are denoted by horizontal lines above each comparison.

Figure 3 displays the mean ordinal regression posterior distributions and associated Likert-style responses for items querying people’s perceived risk from COVID-19 in each wave of data collection. Risk items are described in Table 2. Error bars display the 95% HDI and black horizontal lines illustrate differences where HDIs do not overlap. Over time, participants viewed COVID-19 as “somewhat” harmful to others and were “somewhat” concerned for their own health. The virus was perceived as “very” harmful to one’s health and spiked during wave 3, corresponding with 22 infected naval officers disembarking in Taiwan [38]. Although participants were “very” concerned for others, this concern decreased from wave 1 to wave 4.

### 3.4. Perceived Benefits from Tracking

**Figure 4:**
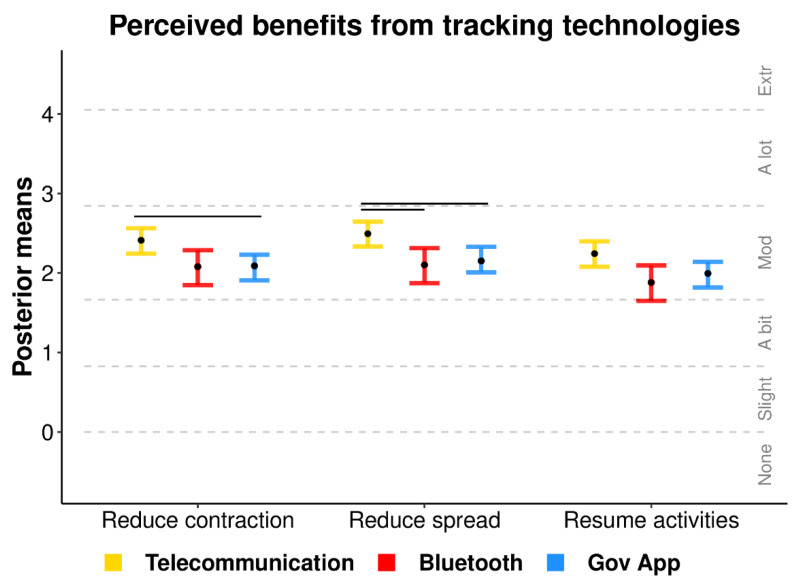
Ordinal regression mean posterior distributions for items assessing the perceived benefits from tracking technologies. Black points display posterior means, error bars display the 95% highest posterior density interval, and dotted lines depict boundaries separating the ordinal responses (none to extremely). Non-overlapping intervals within items are denoted by horizontal lines above each comparison.

Figure 4 displays the mean ordinal regression posterior distributions and associated Likert-style responses for items querying people’s perceived benefits from tracking in each scenario (items described in Table 3). All technologies were perceived to be “moderately” beneficial. Telecommunication network tracking was perceived as the most beneficial for reducing the rate of contraction and spread of COVID-19. All technologies were perceived to show similar benefits for resuming normal activities.

### 3.5. Perceptions of Tracking Technologies

**Figure 5:**
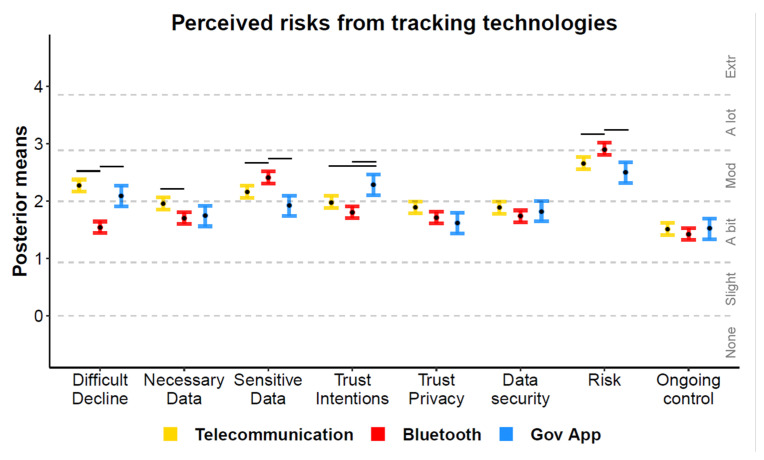
Ordinal regression mean posterior distributions for items assessing the perceived risks from tracking technologies. Black points display posterior means, error bars display 95% highest posterior density intervals, and dotted lines depict boundaries separating ordinal responses; horizontal lines above each comparison denote non-overlapping intervals.

Figure 5 displays the mean ordinal regression posterior distributions and associated Likert-style responses for items querying people’s perceived risks posed by the tracking technologies. Apple and Google’s Bluetooth system was perceived as being the most risky technology, due to collecting more sensitive data than the government options and collecting non-essential data when compared to telecommunication network tracking. However, the Bluetooth system was perceived as easiest to decline participation in. Data privacy, security, and ongoing control were perceived as equivalent across the technologies. The government app was perceived to have the most trustworthy intentions. Usability was not included in the model as it was not assessed for telecommunication tracking; however, both app-based technologies were perceived as “a bit” usable.

### 3.6. Acceptability of Tracking Technologies

**Figure 6:**
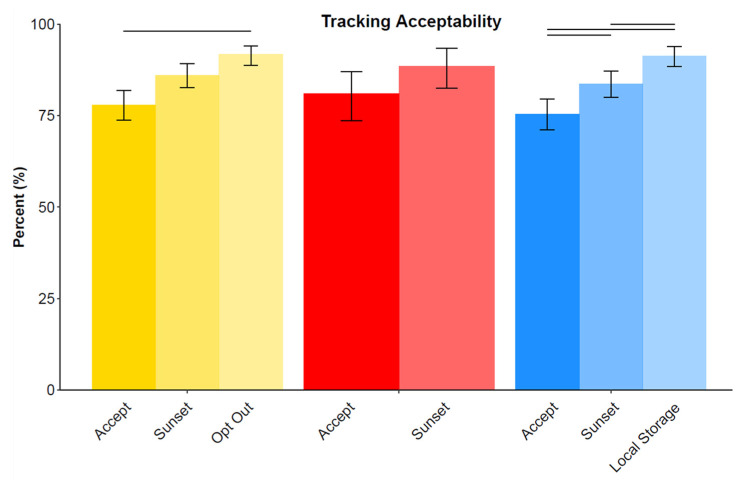
Acceptability for each tracking scenario collapsed across waves, displayed under varying privacy conditions. Error bars are 95% Bayesian credible intervals and non-overlapping intervals within each tracking scenario are denoted by horizontal lines above each comparison.

Figure 6 shows acceptability ratings under varying conditions. Baseline acceptability ratings were measured after responding to the tracking effectiveness items, while the remaining items display the additional acceptance conferred when additional privacy measures were included. Baseline acceptability was very high (75–81%) and did not differ meaningfully across the scenarios. Acceptability increased with additional privacy options in the telecommunication and government app scenarios (up to 92% and 91%, respectively). Acceptability only increased slightly from baseline in the Bluetooth scenario (81% up to 91%) with the inclusion of a sunset clause.

## 4. Discussion

Over the month of April, 2020, we asked four samples of Taiwanese young adults to rate the perceived risks posed by COVID-19, before rating the acceptability, benefits, and risks associated with three hypothetical tracking scenarios: telecommunication network tracking, a decentralized Bluetooth Exposure Notification system backed by Apple and Google, and a centralized government app. Participants perceived COVID-19 as somewhat to very harmful to their health, and were particularly concerned by the risk COVID-19 posed to others. The three tracking technologies were perceived to confer similar benefits to public health and assisting in a return to normal activities; however, they differed in their risks. The Apple/Google EN system was viewed as the most risky and as collecting the most sensitive data; however, this technology was perceived as the easiest to decline participation in and had similar privacy and security ratings to the government alternatives which were generally viewed as more trustworthy and as only collecting necessary data. Regardless of these differences, acceptance was very high for all tracking scenarios (>75% at baseline) and surpassed 90% acceptance once additional privacy measures were included. 

### 4.1. Policy Implications

Our results indicate that the introduction of mobile tracking technologies by the Taiwanese government or trusted corporate entities, such as Google and Apple, would be supported by young Taiwanese adults. Results reflect attitudes towards tracking technologies when COVID-19 cases were approaching zero in Taiwan, and therefore, may be considered as a baseline for COVID-19 tracking acceptance. Given the privacy calculus (i.e., the weighing of risks and benefits when disclosing sensitive information), if cases were to increase in Taiwan, we expect acceptance would similarly increase.

Acceptance was very high for all proposed tracking technologies and the technologies did not differ greatly in their perceived benefits or risks to privacy and security. However, participants displayed great concern for the health of others. These results suggest young Taiwanese adults have a strong sense of communal responsibility to the public’s health, and heavily weigh the public health benefits of mobile tracking technologies against the personal risks to privacy and security. However, this is not to say that privacy considerations did not impact participants’ decision making.

Acceptability increased in each scenario with the inclusion of additional privacy preserving measures, such as a sunset clause, opt-out option, or local storage option. This suggests participants were actively weighing the risks and benefits of the tracking technologies and engaging in privacy calculus. Furthermore, these results suggest that policy makers who seek to increase tracking technology acceptance should not highlight the technology itself (e.g., Bluetooth, telecommunication, GPS), and should instead highlight the privacy measures that accompany these technologies, as well as factors external to the privacy calculus, such as usability and ongoing control over the data.

One factor of the Health Belief Model not addressed in our survey is the importance of “calls to action” that encourage the public to engage in a health policy. For messaging to be effective, policy makers must know what issues to purse in order to affect public behaviors. Our findings show that as the threat from COVID-19 waned, concern for others remained very high. This indicates that proactive public actions among young adults that foster self-efficacy, such as downloading a tracking app, would be best incentivized when framed as “protecting those most at risk”. Furthermore, online and print newspapers and social media were the primary sources for COVID-19 information. We suggest government advertising should focus their attentions on these media to rapidly affect public health behaviors among young adults.

### 4.2. Broader Implications

The current research fits within a broader series of investigations into international acceptance for COVID-19 tracking technologies. Although international results are still being collated, our preliminary findings suggest that both tracking acceptance and concern for others were generally higher among young Taiwanese adults than among representative Australian [31] and United Kingdom [30] samples. Nevertheless, all countries show increasing levels of tracking acceptance once additional privacy measures (e.g., a sunset clause) are included, suggesting that privacy concerns and health surveillance similarly trade-off regardless of culture and age.

These international comparisons must be interpreted with two important caveats: (i) young Taiwanese adults may not be representative of all Taiwanese attitudes and (ii) COVID-19 cases were fewer in Taiwan than in Australia or the United Kingdom. Lower cases in Taiwan would suggest concern and acceptance should be lower than in countries with greater case numbers (e.g., Australia or the United Kingdom), however this was not the case. The elevated concern for others and higher technological acceptance in Taiwan relative to Australia and the United Kingdom may reflect a communal responsibility to public health that is absent in the comparatively individualistic Western cultures. Alternatively, these findings may reflect attitudes common among young adults across countries and cultures, but that appear amplified in Taiwan due to our sampling of young adults. Future research may address these questions by assessing attitudes in a representative sample of Taiwan and comparing attitudes across countries and cultures, while controlling for key demographics, such as age and education.

### 4.3. Limitations

This study aimed to determine attitudes towards tracking technologies among young Taiwanese adults, however, this scope presents a major limitation to our findings. For tracking technologies to be most effective, acceptance must be relatively high among the entire population, not just young adults. Telecommunication network tracking bypasses this issue, working on all devices regardless of the user’s age or attitudes; however, voluntary apps require the user’s consent and self-efficacy to be installed and used effectively. By targeting young adults, we establish acceptance among a critical COVID-19 demographic that may play a role in a second COVID-19 outbreak; however, we also limit the generalizability of our findings. 

Our findings were also limited by our sample size within each wave of data collection, a necessary restriction due to financial constraints and the immediate need for data during the early days of the pandemic. Fortunately, no major changes to the state of the pandemic occurred in Taiwan during our four-week collection window, and public perceptions were relatedly stable, allowing us to pool data across waves to provide an informative analysis. However, future research would benefit by wider time intervals between waves and larger samples therein, to assess how attitudes change over time. Additionally, future research may also aim to establish the acceptability of tracking technologies in a representative sample of the Taiwanese public, as the effectiveness of these technologies among the entire population depends on their uptake among the wider public. 

Finally, our study was limited to assessing three hypothetical tracking scenarios, a small sample of the potential technological solutions now available [39]. By asking about real-world technologies that were under consideration around the world, we explored a range of different scenario dimensions, such as autonomy (mandatory vs. voluntary tracking), storage (centralized vs. decentralized), and data usage (government vs. corporate); however, this study design cannot independently assess the effect of these elements on acceptability judgments. Future research may narrow its focus of investigation to these specific elements to better understand their impact on the participant’s acceptance of privacy-encroaching tracking technologies.

## 5. Conclusions

When sampled through a series of four surveys, we found young Taiwanese adults were very accepting of three hypothetical COVID-19 tracking technologies: a government app, telecommunication network tracking, and the Apple/Google Bluetooth exposure notification system. Acceptance increased further with the inclusion of privacy-preserving measures, such as a sunset clause or local data storage option, and was generally higher than tracking acceptance in Western countries, such as the United Kingdom [30] and Australia [31]. In lieu of a vaccine, these tracking technologies offer one solution that allows for normal social contact among young adults, while ensuring they can be rapidly notified to stop the virus’ spread if they encounter an infected individual. However, for these technologies to be most effective, future research must seek to establish their acceptability among the entire population. We hope that this study can prove informative to decision makers not only in Taiwan, but in other countries with similar cultures and attitudes towards privacy and public health that are attempting to arrest the spread of COVID-19.

## Figures and Tables

**Table 1:** Tracking technologies for COVID-19 and their precision, risk to the user if data were leaked, benefits in COVID-19 tracking, control over when tracking occurs, and who has access to the data. App—mobile phone application.

	Telecommunication Network Tracking	GPS Tracking	Bluetooth Tracking
Precision	50 m–2 km radius due to tower density [24].	5 m radius, but may not work indoors [21].	10 m radius, can be blocked by objects [25].
Risks	Rough locations (e.g., work and home).	Exact locations and movements.	Phone ID is public, but anonymized.
Benefits	Works on all devices. No app needed.	Constant tracking gives location history.	Precision reflects the radius of infection.
Control	Flight mode on or phone off.	Turn GPS or phone off.	Turn Bluetooth or phone off.
Access	Telco company and government.Could be mandatory.	Gov or corporate apps with the user’s permission.	Gov or corporate apps with the user’s permission.

**Table 2:** Items assessing the perceived risk from COVID-19.

Item	Question	Label
Risk 1	How severe do you think novel coronavirus (COVID-19) will be for the general population?	General harm
Risk 2	How harmful would it be for your health if you were to become infected with COVID-19?	Personal harm
Risk 3	How concerned are you that you might become infected with COVID-19?	Concern self
Risk 4	How concerned are you that somebody you know might become infected with COVID-19?	Concern others
Impact 1	Have you ever tested positive to COVID-19?	Positive self
Impact 2	Has somebody you know ever tested positive to COVID-19?	Positive other
Impact 3	How many days, if any, have you been in quarantine or self-isolation?	Lockdown days
Impact 4	Have you temporarily or permanently lost your job as a consequence of the COVID-19 pandemic?	Job loss

**Table 3:** Items assessing the benefits and harm arising from smartphone tracking. “The Government” was replaced by “Apple and Google” in the Bluetooth scenario. Reverse scored items are denoted by [R].

Item	Question	Label
Bfit 1	How confident are you that the described scenario would reduce your likelihood of contracting COVID-19?	Reduce contraction
Bfit 2	How confident are you that the described scenario would help you resume your normal activities more rapidly?	Resume activity
Bfit 3	How confident are you that the described scenario would reduce the spread of COVID-19?	Reduce spread
Harm 1	How difficult is it for people to decline participation?	Difficult to decline [R]
Harm 2	To what extent do people have ongoing control of their data?	Ongoing control
Harm 3	How sensitive are the data being collected?	Data sensitivity
Harm 4	How serious is the risk of harm from the proposed scenario?	Risk (from tracking)
Harm5	How secure are the data that would be collected?	Data security [R]
Harm 6	To what extent is the Government [Apple/Google] only collecting the data necessary to achieve the purposes of the policy?	Data necessary
Harm 7	How much do you trust the Government [Apple/Google] to use the tracking data only to deal with the COVID-19 pandemic?	Trust intentions
Harm 8	How much do you trust the Government [Apple/Google] to be able to ensure the privacy of each individual?	Trust privacy

**Table 4:** Sample sizes, demographics, and information source for COVID-19 in Taiwan for each wave of data collection.

Assessment Item		Wave 1	Wave 2	Wave 3	Wave 4
Initial Sample		385	232	301	169
Removals	Comprehension check	40	33	35	22
Final sample		345	199	266	147
Gender (%)	Men	50.1%	52.8%	50%	53.1%
	Women	49.6%	46.2%	49.6%	46.9%
	Other	-	0.5%	0.4%	-
	Prefer not to say	0.3%	0.5%	-	-
Age (years)	Mean	20.46	19.94	19.8	19.86
	Std. Dev	1.9	1.58	1.35	1.44
Education (%)	Less than high school	0.9%	4.5%	1.9%	3.4%
	Graduated high school	66.1%	76.4%	78.2%	81%
	Graduated university	33%	19.1%	19.9%	15.6%
Information sources	Newspaper	62.3%	68.8%	62%	59.9%
for COVID-19 (%)	Social media	29.9%	23.6%	32%	35.4%
	Television	5.5%	2.5%	2.6%	2%
	Friends and family	1.2%	4%	3%	2.7%
	Other	1.2%	1%	0.4%	-

## Data Availability

The data, code and surveys presented in this study are openly available through the Open Science Foundation (OSF) repository: https://osf.io/tcqae.

## Appendix A. Resilience and World View

The following appendix displays results to survey items that were deemed supplemental to the main investigation. We display these in the interest of transparency to the reader, however, note that these results did not meaningfully contribute to the conclusions displayed in the main study. In summary, psychological resilience and political worldview did not play an important role in the acceptance of tracking technologies, potentially due to the homogeneity of the sample and generally high levels of resilience.

### Appendix A.1. Resilience

After providing consent and demographic information, participants completed the Connor–Davidson resilience scale (CD-RISC). The CD-RISC is a 25-item scale designed to measure how well people deal with the adversity of stressful events, tragedy, and trauma. In Connor and Davidson’s initial study [32], the scale displayed a high degree of internal consistency (Cronbach’s alpha = 0.89) and test–retest reliability (Pearson’s *r* = 0.97). The items sum to provide a measure of psychological resilience.

### Appendix A.2. World View

At the conclusion of each survey, participant’s world views were examined using three items on a 7-point scale, (e.g., 1 = Strongly agree, 7 = Strongly disagree), measuring the endorsement of small governments and the utility of the free market (greater agreement indicated more conservative/libertarian worldviews). These items are summarized in Table A1.

**Table A1:** Items assessing world view (Wview).

Item	Question	Label
Wview 1	An economic system based on free markets unrestrained by government interference automatically works best to meet human needs.	Free market
Wview 2	The free market system may be efficient for resource allocation but it is limited in its capacity to promote social justice.	Social justice
Wview 3	The government should interfere with the lives of citizens as little as possible.	Small government

### Appendix A.3. Results

The ability to cope with stress and trauma during the pandemic may influence (i) how one perceives the severity of the pandemic, and (ii) the perceived risks and benefits of using tracking technologies. Indeed, recent research suggests resilience is associated with less concern about the pandemic and lower level of generalized anxiety and depression [40], which may influence whether tracking technologies are perceived to be necessary or beneficial to public health.

Resilience was measured by the Connor–Davidson resilience scale which ranges from 0 (no resilience) to 100 (high resilience). Resilience scores were moderate to high (M = 62, SD = 12), suggesting participants were psychologically capable of dealing with the stresses and changes associated with the COVID-19 pandemic. We conducted an exploratory analysis and correlated our measure or resilience with items measuring COVID-risk, tracking benefits, tracking risks, and tracking acceptance. Results showed small significant (Bonferroni corrected p’s < 0.001) Pearson’s correlations between psychological resilience and the perception that tracking technologies will reduce the likelihood of contraction, aid in a return to normal activities, and reduce the virus’ spread (*r* = 0.15, 0.19, and 0.17, respectively). Resilience similarly correlated with trust that data privacy and data security would be preserved, and the perception of ongoing control over one’s data (*r* = 0.12, 0.13, and 0.1, respectively). The same results were observed using Spearman correlations and no significant correlations were observed between tracking acceptability and psychological resilience.

Libertarian political worldviews, such as the endorsement of small governments and the free market, may be correlated negatively with trust in one’s government and the acceptability of tracking technologies. We find weak significant (Bonferroni correct p’s < 0.01) negative correlations between libertarian worldviews and concern for others and one’s self from COVID-19 (*r* = −0.13 and −0.18, respectively), and acceptance for tracking technologies (*r* = −0.13). The same results were found using Spearman correlations.

## Appendix B. Scenario Descriptions

### Appendix B.1. Chinese Version (Translated; as Presented in the Experiment)

The government app scenario was described as:

“流行病COVID-19 已經迅速地威脅了全球人類的健康。為了減少對醫療保健系統、經濟的影響，並挽救許多生命，「如何限制病毒傳播」是當前最重要的議題。台灣政府可能考慮使用智慧型手機定位追蹤數據，用來識別和聯繫那些可能已經接觸過COVID-19患者的人。用此定位追蹤技術可以辨識出具有高風險的族群並精準地給予治療，能夠降低社區傳染的風險。但不是所有台灣人都需要參與此計畫，而是只有下載政府提供的應用程式（手機APP），並同意進行追蹤和聯繫的人，才會被包含在該計畫中。因此，下載和使用此應用程式的人越多，政府就能更加有效地限制COVID-19的傳播。而這些定位追蹤的數據將以加密格式儲存在安全的伺服器上，只有台灣政府能讀取該伺服器資料。同時，這些資料僅能用於聯繫可能暴露在COVID-19感染風險下的民眾，不會另做其他用途。”

The telecommunication network tracking scenario was described as:

“流行病COVID-19已經迅速地威脅了全球人類的健康。為了減少對醫療保健系統、經濟的影響，並挽救許多生命，「如何限制病毒傳播」是當前最重要的議題。台灣政府可能考慮使用電信公司提供的手機定位追蹤數據，用來識別和聯繫那些可能已經接觸過COVID-19患者的人。用此定位追蹤技術可以辨識出具有高風險的族群並精準地給予治療，能夠降低社區傳染的風險。這個計畫是強制性的，只要你有手機就會被納入計畫當中，而且無法退出。這些定位追蹤的數據將以加密格式儲存在安全的伺服器上，只有台灣政府才能讀取該伺服器資料。必要時，台灣政府可以使用該數據，找到違反隔離或封鎖命令的民眾，並且進行罰款和逮捕。同時，這些數據還將用於通知適當的公共衛生單位進行應對措施，並與可能接觸過COVID-19感染者的民眾進行聯繫，甚至可以基於此數據，制訂個人化的的隔離方式。”

And the Bluetooth (Apple/Google API) scenario was described as:

“流行病COVID-19已經迅速地威脅了全球人類的健康。為了減少對醫療保健系統、經濟的影響，並挽救許多生命，「如何限制病毒傳播」是當前最重要的議題。Apple和Google已提議在既有智慧型手機上增加接觸史追蹤功能，以告知人們是否曾經接觸過COVID-19患者。這將可以讓大眾主動地自我隔離，進而減少COVID-19的社區傳播。當兩人靠近時，他們的手機就會透過藍芽連結。如果有一個人在之後被診斷為感染者，曾經與該名患者有近距離接觸的人，將會被通知，但是政府並不知道這些人是誰。使用此接觸史追蹤功能是完全自主的。被通知的人們將不會知道是誰的篩檢結果呈現陽性。”

### Appendix B.2. English Version (Translated; not Presented in the Experiment)

The government app scenario was described as:

“The COVID-19 pandemic has rapidly become a worldwide threat. Containing the virus’ spread is essential to minimize the impact on the healthcare system, the economy, and save many lives. The Taiwanese Government might consider using smartphone tracking data to identify and contact those who may have been exposed to people with COVID-19. This would help reduce community spread by identifying those most at risk and allowing health services to be appropriately targeted. Only people that downloaded a government app and agreed to be tracked and contacted would be included in the project. The more people that download and use this app the more effectively the Government would be able to contain the spread of COVID-19. Data would be stored in an encrypted format on a secure server accessible only to the Taiwanese Government. Data would only be used to contact those who might have been exposed to COVID-19.”

The telecommunication network tracking scenario was described as:

“The COVID-19 pandemic has rapidly become a worldwide threat. Containing the virus’ spread is essential to minimize the impact on the healthcare system, the economy, and save many lives. The Taiwanese Government might consider using phone tracking data supplied by telecommunication companies to identify and contact those who may have been exposed to people with COVID-19. This would help reduce community spread by identifying those most at risk and allowing health services to be appropriately targeted. All people using a mobile phone would be included in the project, with no possibility to opt-out. Data would be stored in an encrypted format on a secure server accessible only to the Taiwanese Government who may use the data to locate people who were violating lockdown orders and enforce them with fines and arrests where necessary. Data would also be used to inform the appropriate public health response and to contact those who might have been exposed to COVID-19, and individual quarantine orders could be made on the basis of this data.”

And the Bluetooth (Apple/Google API) scenario was described as:

“The COVID-19 pandemic has rapidly become a worldwide threat. Containing the virus’ spread is essential to minimize the impact on the healthcare system, the economy, and save many lives. Apple and Google have proposed adding a contact tracing capability to existing smartphones to help inform people if they have been exposed to others with COVID-19. This would help reduce community spread of COVID-19 by allowing people to voluntarily self-isolate. When two people are near each other, their phones would connect via Bluetooth. If a person is later identified as being infected, the people they have been in close proximity to are then notified without the government knowing who they are. The use of this contact tracing capability would be completely voluntary. People who are notified would not be informed who had tested positive.”

## References

[1] Cheng H.-Y., Li S.-Y., Yang C.-H. (2020). Initial rapid and proactive response for the COVID-19 outbreak—Taiwan’s experience. J. Formos. Med. Assoc..

[2] Milne G.J., Xie S. (2020). The effectiveness of social distancing in mitigating COVID-19 spread: A modelling analysis. medRxiv.

[3] Wang C.J., Ng C.Y., Brook R.H. (2020). Response to COVID-19 in Taiwan: Big data analytics, new technology, and proactive testing. JAMA.

[4] Roser M., Ritchie H., Ortiz-Ospina E., Hasell J. (2020). Coronavirus Pandemic (COVID-19): Our World in Data. https://ourworldindata.org/coronavirus/country/taiwan?country=~TWN.

[5] Byambasuren O., Cardona M., Bell K., Clark J., McLaws M.-L., Glasziou P. (2020). Estimating the extent of true asymptomatic COVID-19 and its potential for community transmission: Systematic review and meta-analysis. Off. J. Assoc. Med. Microbiol. Infect. Disease Can..

[6] World Health Organization Virtual Press Conference on COVID-19 in the Western Pacific: Remarks by Dr Takeshi Kasai. https://www.who.int/westernpacific/news/speeches/detail/virtual-press-conference-on-covid-19-in-the-western-pacific.

[7] Liao J., Fan S., Chen J., Wu J., Xu S., Guo Y., Li C., Zhang X., Wu C., Mou H. (2020). Epidemiological and clinical characteristics of COVID-19 in adolescents and young adults. Innovation.

[8] Laxminarayan R., Wahl B., Dudala S.R., Gopal K., Mohan C., Neelima S., Reddy K.S.J., Radhakrishnan J., Lewnard J. (2020). Epidemiology and transmission dynamics of COVID-19 in two Indian states. Science.

[9] Johnson C., Lyons S. Superspreaders and the Role They Play in Transmitting Coronavirus to Others. https://www.abc.net.au/news/health/2020-07-15/superspreader-events-people-places/12457542.

[10] Kucharski A.J., Klepac P., Conlan A., Kissler S.M., Tang M., Fry H., Gog J.R., Edmunds W.J. (2020). Effectiveness of isolation, testing, contact tracing and physical distancing on reducing transmission of SARS-CoV-2 in different settings. Lancet Infect. Dis.

[11] Oliver N., Lepri B., Sterly H., Lambiotte R., Deletaille S., De Nadai M., Letouzé E., Salah A.A., Benjamins R., Cattuto C. (2020). Mobile phone data for informing public health actions across the COVID-19 pandemic life cycle. Am. Assoc. Adv. Sci..

[12] Ferretti L., Wymant C., Kendall M., Zhao L., Nurtay A., Abeler-Dörner L., Parker M., Bonsall D., Fraser C. (2020). Quantifying SARS-CoV-2 transmission suggests epidemic control with digital contact tracing. Science.

[13] Boutilier R.G., Thomson I. (2020). Modelling and measuring the social license to operate: Fruits of a dialogue between theory and practice. Soc. Licence.

[14] Laufer R.S., Wolfe M. (1977). Privacy as a concept and a social issue: A multidimensional developmental theory. J. Soc. Issues.

[15] Culnan M.J., Armstrong P.K. (1999). Information privacy concerns, procedural fairness, and impersonal trust: An empirical investigation. Organ. Sci..

[16] Wang T., Duong T.D., Chen C.C. (2016). Intention to disclose personal information via mobile applications: A privacy calculus perspective. Int. J. Inf. Manag..

[17] Sun Y., Wang N., Shen X.-L., Zhang J.X. (2015). Location information disclosure in location-based social network services: Privacy calculus, benefit structure, and gender differences. Comput. Hum. Behav..

[18] Braithwaite I., Callender T., Bullock M., Aldridge R.W. (2020). Automated and partly automated contact tracing: A systematic review to inform the control of COVID-19. Lancet Digit. Health.

[19] Hochbaum G.M. (1958). Public Participation in Medical Screening Programs: A Socio-Psychological Study (No. 572).

[20] Llewellyn C., Ayers S., McManus C., Newman S.P., Petrie K., Revenson T., Weinman J. (2019). Cambridge Handbook of Psychology, Health and Medicine.

[21] United States Air Force GPS Accuracy. https://www.gps.gov/systems/gps/performance/accuracy/#:~:text=Forexample,GPS-enabledsmartphones,receiversand/oraugmentationsystems.

[22] Lee Y. (2020). Taiwan’s new ‘electronic fence’ for quarantines leads wave of virus monitoring. Reuters Technology News.

[23] Tsou H.-H., Cheng Y.-C., Yuan H.-Y., Hsu Y.-T., Wu H.-Y., Lee F.-J., Hsiung C.A., Chen W.J., Sytwu H.-K., Wu S.-I. (2020). The effect of preventing subclinical transmission on the containment of COVID-19: Mathematical modeling and experience in Taiwan. Contemp. Clin. Trials.

[24] Trogh J., Plets D., Surewaard E., Spiessens M., Versichele M., Martens L., Joseph W. (2019). Outdoor location tracking of mobile devices in cellular networks. EURASIP J. Wirel. Commun. Netw..

[25] Samsung What Is the Maximum Range of a Bluetooth Connection?. https://www.samsung.com/levant/support/mobile-devices/what-is-the-maximum-range-of-a-bluetooth-connection.

[26] Google Exposure Notifications: Using Technology to Help Public Health Authorities Fight COVID-19. https://www.google.com/covid19/exposurenotifications.

[27] Katherine-Chen Y.-N., Ryan-Wen C.-H. (2019). Taiwanese university students’ smartphone use and the privacy paradox. Comun. Media Educ. Res. J..

[28] Ali A.J., Lee M., Hsieh Y.-C., Krishnan K. (2005). Individualism and collectivism in Taiwan. Cross Cult. Manag..

[29] Viens A. (2016). Public health and political theory: The importance of taming individualism. Public Health Ethics.

[30] Lewandowsky S., Dennis S., Perfors A., Kashima Y., White J.P., Garrett P.M., Little D.R., Yesilada M. (2021). Public acceptance of privacy-encroaching policies to address the COVID-19 pandemic in the United Kingdom. PLoS ONE.

[31] Garrett P.M., White J.P., Lewandowsky S., Kashima Y., Perfors A., Little D.R., Geard N., Mitchell L., Tomko M., Dennis S. (2021). The acceptability and uptake of smartphone tracking for COVID-19 in Australia. PLoS ONE.

[32] Connor K.M., Davidson J.R. (2003). Development of a new resilience scale: The Connor-Davidson resilience scale (CD-RISC). Depress. Anxiety.

[33] Martin A.D., Quinn K.M., Park J.H. (2011). Mcmcpack: Markov chain monte carlo in R. J. Stat. Softw..

[34] Plummer M., Best N., Cowles K., Vines K. (2006). Coda: Convergence diagnosis and output analysis for mcmc. R. News.

[35] Albert J.H., Chib S. (1993). Bayesian analysis of binary and polychotomous response data. J. Am. Stat. Assoc..

[36] Bürkner P.-C., Vuorre M. (2019). Ordinal regression models in psychology: A tutorial. Adv. Methods Pract. Psychol. Sci..

[37] Bååth R. (2014). Bayesian first aid: A package that implements Bayesian alternatives to the classical*. test functions in r. Proc. UseR.

[38] Davidson H. Taiwan Navy Races to Trace 700 Sailors on Pacific ’Goodwill’ Tour after Covid-19 Cases Emerge. https://www.theguardian.com/world/2020/apr/22/taiwan-navy-races-to-trace-700-sailors-on-pacific-goodwill-tour-after-covid-19-cases-emerge.

[39] O’Neill P., Ryan-Mosley T., Johnson B. A Flood of Coronavirus Apps are Tracking us. Now It’s Time to Keep Track of Them. https://www.technologyreview.com/2020/05/07/1000961/launching-mittr-covid-tracing-tracker.

[40] Barzilay R., Moore T.M., Greenberg D.M., Di Domenico G.E., Brown L.A., White L.K., Gur R.C., Gur R.E. (2020). Resilience, COVID-19-related stress, anxiety and depression during the pandemic in a large population enriched for healthcare providers. Transl. Psychiatry.

